# One-Class Classification by Ensembles of Random Planes (OCCERPs)

**DOI:** 10.1155/2022/4264393

**Published:** 2022-07-04

**Authors:** Amir Ahmad

**Affiliations:** College of Information Technology, United Arab Emirates University, Al-Ain, UAE

## Abstract

One-class classification (OCC) deals with the classification problem in which the training data have data points belonging only to the target class. In this paper, we present a one-class classification algorithm, One-Class Classification by Ensembles of Random Plane (OCCERP), that uses random planes to address OCC problems. OCCERP creates many random planes. There is a pivot point in each random plane. A data point is projected in a random plane and a distance from a pivot point is used to compute the outlier score of the data point. Outlier scores of a point computed using many random planes are combined to get the final outlier score of the point. An extensive comparison of the OCCERP algorithm with state-of-the-art OCC algorithms on several datasets was conducted to show the effectiveness of the proposed approach. The effect of the ensemble size on the performance of the OCCERP algorithm is also studied.

## 1. Introduction

The one-class classification (OCC) problem is a special class of classification problems in which only the data points of one class (the target set) are available [1]. The task in one-class classification is to make a model of a target set of data points and to predict if a testing data point is similar to the target set. The point which is not similar to the target set is called an outlier. OCC algorithms have applications in various domains [2] including anomaly detection, fraud detection, machine fault detection, and spam detection [2].

The OCC problem is generally considered to be a more difficult problem than the two-class classification problem as the training data have only data points belonging to one class [1–3], and traditional classifiers need training data from more than one class to learn decision boundaries. Therefore, standard classifiers cannot be applied directly to OCC problems. Various algorithms have been proposed to address OCC problems [1–3].

There are two main approaches to handle OCC problems [2, 3]. In the first approach, artificial data points for the nontarget class (outlier) are generated and combined with the target data points and then a binary classifier is trained on this new data. In the second approach, target data points are used to create the OCC models [4].

Gaussian models [3], reconstruction-based methods [1–3], nearest neighbours [1, 5], support vector machines [6, 7] clustering based methods [1], and convex hull [8] are some examples of the second approach.

Ensembles of accurate and diverse models generally perform better than individual members of ensembles [9]. Ensembles of classification models have been developed to improve the performance of one-class classification models [5, 10–12].

In this paper, we propose an ensemble method, OCCERP, for OCC problems. In this method, we project data points in a random plane. The distance of a data point from a pivotal point in this random plane is used as an outlier score. We can generate various diverse models by selecting different random planes which can be used to create ensembles. Experiments are done to show the effectiveness of the proposed approach.

The paper is organised as follows: Section 2 discusses about related work. The OCCER algorithm is presented in Section 3.  Section 4 presents experiments and discussion.  Section 5 discusses the conclusion and suggests future developments.

## 2. Literature Survey

As previously discussed there are two types of OCC algorithms, and OCCERP belongs to the second type, which will be discussed in this section.

Generative methods are useful for OCC as the target class may directly be modelled from the available training target data points. Density-based methods, such as Gaussian, kernel density estimators, Parzen windows, and mixture models are widely used for OCC problems [3, 13]. Density-based methods estimate the probability density function of the underlying distribution of the training target data points. Then, these methods determine if a new data point comes from the same distribution. The selection of appropriate models and large-scale training data are the problems of this approach.

Nearest neighbour-based (NN-based) approaches are other widely used methods to address OCC problems [1, 3, 5]. This approach assumes that an outlier point will be far away from neighbour target points as compared to a target point from other neighbour target points [1, 5].

The local outlier factor (LOF) method is a density-based scheme for OCC [14], in which a LOF is computed for each data point by taking the ratios of the local density of the point and the local densities of its neighbours. An outlier point has a large LOF score.

Tax and Duin [15] propose the support vector domain description method for OCC. The method finds a hyper-sphere with a minimum volume around the target class data such that it encloses almost all the points in the target class dataset. Scholkopf et al. [7] propose the use of support vector machines for one class classification. A hyperplane is constructed such that it separates all the data points from the origin and the hyperplane's distance from the origin is maximised.

In reconstruction-based methods [1–3, 16, 17], a model like an autoencoder is trained on the given target class data. The reconstruction error which depends on a testing data point and the system output is used to define the outlier score. An outlier point is likely to have more reconstruction errors.

Clustering-based approaches use a clustering method, like k-means clustering to create clusters [1]. The distance between a data point and its nearest cluster centre is used as the outlier score. The number of clusters and cluster initialization are the problem of k-means type clustering algorithms.

Rahimzadeh Arashloo and Kittler [18] present a nonlinear one-class classifier formulated as the Rayleigh quotient criterion optimisation that projects the target class points to a single point in a new feature space, the distance between the projection of a testing point to that point is the outlier score of the testing point. Leng et al. [19] use a similar approach but use extreme learning machines for the projection.

Ensembles have also been developed for the OCC problems. There are two approaches for creating ensembles. In the first approach, one OCC algorithm is employed and a randomisation process is used to create diverse OCC models. Lazarevic and Kumar [20] propose the creation of multiple datasets by using feature bagging. The LOF algorithm is then used on these multiple datasets, hence multiple OCC models are created. The outputs of these models are combined to get the final output. Khan and Ahmad [5] use random projection to create multiple datasets. An NN-based OCC algorithm is applied to these multiple datasets. Arthur et al. [21] introduce noise to the dataset to create multiple datasets. Experiments with different OCC algorithms show the effectiveness of the proposed approach. Chen et al. [11] use randomly connected autoencoders to create ensembles of autoencoders. These ensembles outperformed other state-of-the art OCC methods. Khan and Taati [22] train different autoencoders using different features to create ensembles of autoencoders. They show that ensembles perform better than single autoencoders. Isolation forests consist of many decision trees [10]. These trees are created by using random partitioning. The authors argue that anomalies are susceptible to isolation and therefore have short path lengths. The method has produced excellent results on various datasets. Kanag [23] uses the clustering technique to many clusters. Therese clusters are used using the one-against-rest method to create many binary-classifiers. Their classifiers are used as an ensemble to handle OCC problems. Mohammeda and Kora [24] propose ensembles of deep learning models for text classification problems.

## 3. One Class Classification by Ensembles of Random Planes (OCCERPs)

For OCC problems, the training data have points from one class. In this section, we will call this class as the negative class. A class consisting of outlier points will be called as the positive class. The motivation of the proposed approach is that if data points are projected on a plane, the distance from a properly selected pivot point to the projection of a given point can be used as an outlier score. The projections of negative class points are expected to be nearer to this pivotal point as compared to the projections of positive class points. Many random planes can be generated. Each plane will generate one outlier score for a given point, and all the scores will be combined to get the final outlier score for a point.

**Figure 1:**
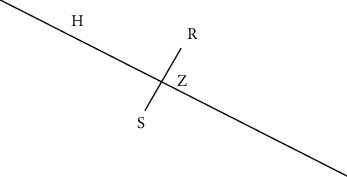
A plane, H created by two points using two data points *R* and *S*. The plane is the random plane that will pass from the midpoint, *Z* of *R* and *S* points and will have the normal going through these two data points.

Creating appropriate random planes and selecting appropriate pivotal points on these random planes are very important steps of the proposed approach. We use the random linear oracle approach [25] to create random planes and pivotal points. Kuncheva and Rodriguez [25] propose a random linear oracle approach for classifier ensembles. In this approach, they divide the training data points into two groups using a random linear oracle (RLO). This RLO is a random hyperplane which is created by using two randomly selected points from the training data. We use the same approach to generate random planes. To create a random plane, two points are randomly selected from the given negative class. The random plane will pass from the midpoint of the two selected data points and will have the normal going through these two data points (Figure 1). As these two data points are part of the negative class, the midpoint is expected to be within the boundary of the negative class. This point will act as a pivotal point to compute the outlier score of a given data point. RLO approach makes sure that there are points on both sides of the hyperplane.

We will discuss the mathematical formulas used in the proposed approach. In a *n* dimension space (*X*_1_, *X*_2_, *X*_3_,…, *X*_*n*_), two randomly selected points are *R*(*r*_1_, *r*_2_, *r*_3_,…, *r*_*n*_) and *S*(*s*_1_, *s*_2_, *s*_3_,…, *s*_*n*_).

The equation of the plane in *n* dimension is

(1) $$\begin{array}{c} A_{1}X_{1}+A_{2}X_{2}+A_{3}X_{3}+\cdots +A_{n}X_{n}+B=0, \end{array}$$

where *A*_1_, *A*_2_, *A*_3_,…, *A*_*n*_ are directions and *B* is a constant.

The values of *A*_*i*_ and B of a plane for which the normal is going through two points *R* and *S* and a point *Z*(*z*_1_, *z*_2_, *z*_3_,…, *z*_*n*_) is on the plane

(2) $$\begin{array}{c} A_{i}=r_{i}-s_{i}B=-\left(A_{1}z_{1}+A_{2}z_{2}+A_{3}z_{3}+\cdots +A_{n}z\right). \end{array}$$

In random linear oracle, the plane goes from the midpoint of *X* and *Y*, therefore *Z* is defined as follows:

(3) $$\begin{array}{c} z_{i}=\frac{\left(r_{i}+s_{i}\right)}{2}. \end{array}$$

The perpendicular distance *D*_1_ from a point *P*_1_(*p*_1_, *p*_2_, *p*_3_,…, *p*_*n*_)

(4) $$\begin{array}{c} D_{1}=\frac{\sum_{1}^{n}A_{i}p_{1}+B}{\sum_{1}^{n}{\left(A_{i}\right)}^{2}}. \end{array}$$

The distance between the point *P* and. *ZD*_2_=∑_1_^*n*^(*p*_*i*_ − *z*_*i*_)^2^

**Figure 2:**
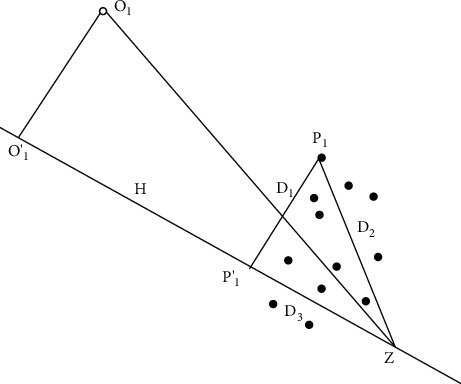
*P*_1_′ and *O*_1_′ are the projected points of *P*_1_ and *O*_1_ on the plane H. Z is the pivotal point. *P*_1_′*Z* is the outlier score of point *P*_1_′, and *O*_1_′*Z* is the outlier score of point *O*_1_′. The outlier score of the positive point is larger.

From Figure 2.(*D*_3_)^2^=(*D*_2_)^2^ − (*D*_1_)^2^

*D*_3_ will be used as an outlier score.

Figure 2 shows that *D*_3_ will be small negative class points whereas this value will be large for positive class points.

### 3.1. Combination of Results

Researchers use different approaches to combine the results of different outlier models such as mean, median, maximum, and minimum [26, 27]. There is no proper justification in literature for selecting one over the other. We did a small experiment with five-fold cross validation with three datasets to understand their performances. We found that there is no approach which performed consistently best for all the runs. However, we found that the minimum approach has an advantage over other approaches. Therefore, we selected the minimum approach to combine the results. To avoid the effect of extreme value, instead of minimum value, we took the mean of five minimum values. All the experiments were done using this combination approach. We did not experiment with numbers other than five. It is noted that the combination of different outlier models in an ensemble is an important research problem. We do not claim that the minimum approach is best. This research problem requires more experimental and theoretical analysis which is beyond the scope of this paper.

## 4. Experiments

We conducted experiments by using the scikit-learn python package (https://scikit-learn.org/stable/) and PyOD (a Python toolbox for scalable outlier detection) [30]. Different standard OCC algorithms, Isolation Forests (IFs), One-class SVM (OCSVM), LOF, and autoencoders were used for the comparative study. PyOD was used for these methods. The default parameters for these methods given in PyOD were used in the experiments. For the OCCERP algorithm, the same size and the fixed combination approach was used. 5 × 2-fold cross-validation was used for the experiments. Stratified k-fold was implemented using scikit-learn to ensure that the folds were made by preserving the percentage of the samples for each class. Only the negative class points in the training data were used to train the OCC algorithms. z-normalisation was used to normalise the data. As classification accuracy is not a correct performance matrix due to the highly-imbalanced testing data, we used the average area under the curve (AUC) for the receiver operating characteristics(ROCs) curve as it is generally used to measure the performance of OCC algorithms [10, 11]. We carried out experiments with the OCCERP algorithm with 500 random planes (OCCERP (500)). We applied a statistical test, the Sign test [31] to compare the performance of OCCERP (500) against other one-class classifiers. It is based on counts of wins, losses, and ties. If the number of wins is at least *N*/2 + $1.96\sqrt{n}/2$, the classifier is significantly better with *p* < 0.05. In our experiments, the total number of datasets is 26, therefore if the number of wins is 18, the classifier is statistically better than the other classifier.

### 4.1. Standard Datasets and Domain Datasets

Various kinds of datasets were used in the experiments [28, 29, 32–35], Some datasets are created as imbalanced datasets [28, 29]. Information on these datasets is presented in Table 1. The domain datasets [32–35] belong to two different domain datasets: normal activity-fall activity datasets and software engineering-related datasets. The domain datasets [32–35] are naturally imbalanced datasets. Mobilfall data [32] were collected using Samsung Galaxy S3 mobile employing the integrated 3D accelerometer and gyroscope. The data have two classes normal activity and fall activity. We used the data collected from 11 subjects who performed various normal and fall activities. We grouped the German Aerospace Centre (DLR) data [33] into normal activity and fall activity and only used the data from the accelerometer and gyroscope. Only data from those subjects who performed both the activities were used. Coventry dataset (COV) [34] also has two classes normal activity and fall activity, and the complete information of these domain datasets is presented in detail in [5]. Information on these domain datasets is presented in Table 2.

Software engineering-related datasets were taken from NASA's metrics data program data repository. This repository has defect data of various software projects written using different programming languages. cm1 and pc1 are written in C. kc1 and kc2 are implemented using C++. Datatrieve is composed of C functions and BLISS subroutines. class-level-kc1-defect-or-not and class-level-kc1-defect-count-ranking use only larger modules of kc1 data. class-level-kc1-defect-count-ranking data has two classes based on if the defects are in the top 5% in defect ranking or not. The software projects are described using different features such as McCabe measures [36] and Halstead measures [37]. Information on these software engineering-related datasets is presented in Table 2.

### 4.2. Results

The results (average AUCROC) for datasets presented in Table 1 are provided in Table 3, which suggest that out of 16 datasets, OCCERP (500) performed best for eleven datasets. LOF method performed best for eight datasets. Both achieved the joint best results for three datasets.

The results (average AUC) for domain datasets (presented in Table 2) are provided in Table 4. The OCCERP (500) performed best for seven datasets out of ten datasets, whereas other OCC algorithms were best for three datasets. If performed best for two datasets, whereas LOF performed best for one dataset. The results suggest the superior performance of OCCERP (500) over other standard OCC algorithms.

Wins, losses, and ties for OCCERP (500) against other OCC algorithms for all 26 datasets are presented in Table 5. As discussed earlier, if the win is equal to or more than 18 the OCCERP (500) is statistically better than that algorithm. The number of wins is at least 18 for OCCERP (500) against all other OCC algorithms. This shows that OCCERP (500) is statistically better than other OCC algorithms.

### 4.3. Effect of Size of OCCERP Ensembles

OCCERP is an ensemble of many OCC models. An ensemble is accurate if it consists of many accurate and diverse models. If models are diverse, the performance of an ensemble improves with the size. To study the effect of the size on the performance of the OCCERP algorithm, we created OCCERP with 200 random planes (OCCERP (200)) and compared OCCERP (200) with (OCCERP (500)). The results are presented in Tables 6 and 7. For most of the datasets, OCCERP (500) performs better than OCCERP (200). It suggests that more models are useful for OCCERP. It shows that OCCERP is able to create diverse OCC models. OCC models are based-on random planes. RLO creates random planes which in turn generate diverse OCC models.

## 5. Conclusion

OCC is a challenging task due to the absence of the outlier class data points in the training dataset. In this paper, we presented OCCERP to address OCC problems. OCCERP creates many OCC models. In each model, a random plane and a pivot point are used to compute an outlier score for a given data point. Outlier scores for the data point are combined using a novel minimum approach. Experiments suggested that OCCERP performed better than or similar to other OCC methods. This shows the effectiveness of the OCCERP method.

In this paper, the RLO approach is used to create random planes and pivot points. In the future, we will develop other approaches to generate random planes and pivot points. The combination of OCCERP with other ensemble approaches, such as bagging [38] (to create different training datasets), is another future research direction. We will also study the performance of OCCERP in the feature space created by random projections and principal component analysis.

## Figures and Tables

**Algorithm 1:**
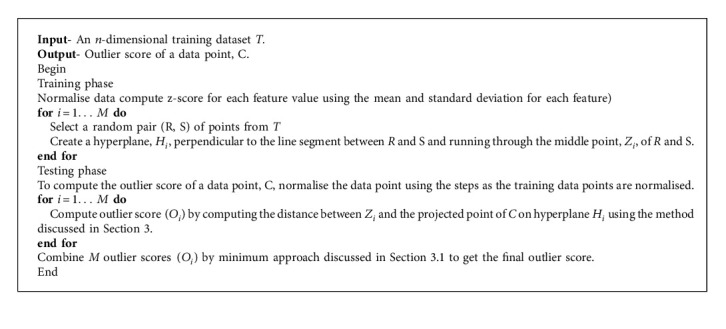
OCCER algorithm.

**Table 1:** Information on the datasets that were taken from [28, 29]. The datasets presented before the separating line in the table are taken from [28] whereas the datasets presented after the separating line are taken from [29].

Dataset	Number of features	Number of data points in negative class	Number of data points of positive class
Pima	8	500	268
segment0	19	1979	329
yeast1	8	1055	429
yeast3	8	1321	163
yeast4	8	1433	51
Winequality-red-4	11	1546	53
Winequality-red-8_vs_6	11	638	18
Winequality-white-3_vs_7	11	880	20
Aloi-unsupervised	27	48492	1508
Annthyroid-unsupervised	21	6666	250
Breast-cancer-unsupervised	30	357	10
Letter-unsupervised	32	1500	100
Satellite-unsupervised	36	5025	75
Shuttle-unsupervised	9	45586	878
Speech-unsupervised	400	3625	61
Pen-local-unsupervised	16	6714	10
Pen-global-unsupervised	16	719	90

**Table 2:** Information on the domain datasets.

Dataset	Number of Features	Number of data points in negative class	Number of data points in positive class
MF	31	488	5430
COV	31	908	12392
DLR	31	84	26576
Class-level-kc1-defectornot	94	60	85
kc2	21	105	415
kc1	21	326	1783
cm1	21	49	449
Datatrieve	8	11	119
pc1	21	77	1032
Class-level-kc1-defect-count-ranking	94	8	137

**Table 3:** Average AUCROC of various OCC algorithms against the OCCERP (500) algorithm on various datasets [28, 29] presented in Table 1. Bold numbers indicate the best performance.

Dataset	If	LOF	OCSVM	Autoencoder	OCCERP (500)
Pima	0.731	0.709	0.700	0.648	**0.738**
segment0	0.474	0.815	0.294	0.342	**0.923**
Winequality-red-4	0.584	0.651	0.615	0.609	**0.655**
Winequality-red-8_vs_6	0.667	0.592	0.647	0.681	**0.716**
Winequality-white-3_vs_7	0.849	0.866	0.853	0.851	**0.928**
yeast1	0.543	**0.615**	0.548	0.534	0.589
yeast3	0.673	**0.807**	0.725	0.728	0.788
yeast4	0.734	0.665	0.733	**0.745**	**0.745**
Aloi-unsupervised	0.539	**0.748**	0.549	0.549	0.556
Annthyroid-unsupervised	0.737	**0.907**	0.727	0.702	0.766
Breast-cancer-unsupervised	0.982	**0.985**	0.985	0.982	**0.985**
Letter-unsupervised	0.627	0.862	0.615	0.526	**0.872**
Satellite-unsupervised	0.949	**0.977**	0.937	0.895	**0.977**
Shuttle-unsupervised	0.995	**0.999**	0.996	0.993	**0.999**
Pen-global-unsupervised	0.947	0.957	0.972	0.869	**0.998**
Pen-local-unsupervised	0.778	**0.985**	0.589	0.440	0.966
Best performance	0	8	0	1	11

**Table 4:** Average AUCROC of various OCC algorithms against the OCCERP (500) algorithm on various domain datasets presented in Table 2. Bold numbers indicate the best performance.

Dataset	If	LOF	OCSVM	Autoencoder	OCCERP (500)
MF	0.969	0.890	0.978	0.941	**0.992**
COV	0.831	**0.912**	0.804	0.769	0.883
DLR	0.947	0.988	0.955	0.978	**0.991**
Class-level-kc1-defectornot	0.797	0.762	0.705	0.607	**0.801**
kc2	**0.839**	0.632	0.806	0.754	0.827
kc1	0.792	0.634	0.708	0.634	**0.807**
cm1	0.704	0.661	0.636	0.518	**0.787**
Datatrieve	0.728	0.690	0.692	0.572	**0.753**
pc1	0.697	0.689	0.676	0.599	**0.719**
Class-level-kc1	**0.903**	0.884	0.864	0.780	0.891
-Defect-count-ranking					
Best performance	2	1	0	0	7

**Table 5:** Wins/losses/ties of OCCERP (500) against other OCC algorithms. A tie is split evenly between the two algorithms.

	If	LOF	OCSVM	Autoencoder
Wins/Losses/Ties	24/2/0	17/6/3	26/0/0	25/0/1
Effective number of wins	24	18	26	25

**Table 6:** Average AUCROC of OCCERP(500) and OCCERP(200) on various datasets [28, 29] presented in Table 1. Bold numbers indicate the best performance.

Dataset	OCCERP (200)	OCCERP (500)
Pima	0.736	**0.738**
segment0	0.851	**0.923**
Winequality-red-4	0.592	**0.655**
Winequality-red-8_vs_6	0.686	**0.716**
Winequality-white-3_vs_7	0.901	**0.928**
yeast1	0.572	**0.589**
yeast3	0.755	**0.788**
yeast4	0.723	**0.745**
Aloi-unsupervised	0.554	**0.556**
Annthyroid-unsupervised	0.662	0.766
Breast-cancer-unsupervised	0.953	**0.985**
Letter-unsupervised	0.857	**0.872**
Satellite-unsupervised	0.963	**0.977**
Shuttle-unsupervised	0.997	**0.999**
Pen-global-unsupervised	0.996	**0.998**
Pen-local-unsupervised	0.953	**0.966**

**Table 7:** Average AUCROC of OCCERP(500) and OCCERP(200) on various domain datasets presented in Table 2. Bold numbers indicate the best performance.

Dataset	OCCERP (200)	OCCERP (500)
MF	0.991	**0.992**
COV	0.852	**0.883**
DLR	0.978	**0.991**
Class-level-kc1-defectornot	0.758	**0.801**
kc2	**0.827**	**0.827**
kc1	0.791	**0.807**
cm1	0.757	**0.787**
Datatrieve	**0.797**	0.753
pc1	0.717	**0.719**
Class-level-kc1-defect-count-ranking	0.889	**0.891**

## Data Availability

The data used in this paper are from previously reported studies and datasets, which have been cited. The data are available at https://sci2s.ugr.es/keel/imbalanced.php, http://promise.site.uottawa.ca/SERepository/datasets-page.html, and https://dataverse.harvard.edu/dataset.xhtml?persistentId=doi:10.7910/DVN/OPQMVF.
